# Novel Long Non-coding RNA and LASSO Prediction Model to Better Identify Pulmonary Tuberculosis: A Case-Control Study in China

**DOI:** 10.3389/fmolb.2021.632185

**Published:** 2021-05-25

**Authors:** Zirui Meng, Minjin Wang, Shuo Guo, Yanbing Zhou, Mengyuan Lyu, Xuejiao Hu, Hao Bai, Qian Wu, Chuanmin Tao, Binwu Ying

**Affiliations:** Department of Laboratory Medicine, West China Hospital, Sichuan University, Chengdu, China

**Keywords:** pulmonary tuberculosis, clinically diagnosed pulmonary tuberculosis, prediction model, least absolute shrinkage and selection operator, electronic health record, laboratory findings, web application

## Abstract

**Introduction:**

The insufficient understanding and misdiagnosis of clinically diagnosed pulmonary tuberculosis (PTB) without an aetiological evidence is a major problem in the diagnosis of tuberculosis (TB). This study aims to confirm the value of Long non-coding RNA (lncRNA) n344917 in the diagnosis of PTB and construct a rapid, accurate, and universal prediction model.

**Methods:**

A total of 536 patients were prospectively and consecutively recruited, including clinically diagnosed PTB, PTB with an aetiological evidence and non-TB disease controls, who were admitted to West China hospital from Dec 2014 to Dec 2017. The expression levels of lncRNA n344917 of all patients were analyzed using reverse transcriptase quantitative real-time PCR. Then, the laboratory findings, electronic health record (EHR) information and expression levels of n344917 were used to construct a prediction model through the Least Absolute Shrinkage and Selection Operator algorithm and multivariate logistic regression.

**Results:**

The factors of n344917, age, CT calcification, cough, TBIGRA, low-grade fever and weight loss were included in the prediction model. It had good discrimination (area under the curve = 0.88, cutoff = 0.657, sensitivity = 88.98%, specificity = 86.43%, positive predictive value = 85.61%, and negative predictive value = 89.63%), consistency and clinical availability. It also showed a good replicability in the validation cohort. Finally, it was encapsulated as an open-source and free web-based application for clinical use and is available online at https://ziruinptb.shinyapps.io/shiny/.

**Conclusion:**

Combining the novel potential molecular biomarker n344917, laboratory and EHR variables, this web-based prediction model could serve as a user-friendly, accurate platform to improve the clinical diagnosis of PTB.

## Introduction

Tuberculosis (TB) remains the leading cause of mortality and morbidity worldwide (World Health Organization, 2020). The most common form of TB is pulmonary tuberculosis (PTB), which accounts for about 85% of all TB cases and poses a serious threat to global health (Faksri et al., 2018). Rapid and accurate diagnosis of PTB is a crucial element in the World Health Organization (WHO)’s End TB Strategy (Uplekar et al., 2015). Currently, two ways are used for PTB detection: detection of *Mycobacterium tuberculosis* (MTB) itself or specific biomarkers of the host immune response (Lyu et al., 2021). For the first method, acid-fast bacilli (AFB) in sputum smear microscopy and the cultivation of MTB complex bacteria are still the gold standard, but they suffer from low sensitivity and consume considerable time (Kohlmorgen et al., 2017; Yang et al., 2020). Although the detection of MTB DNA using Gene Xpert or polymerase chain reaction (PCR) can improve the sensitivity and provide quicker results than cultivation to a certain extent, nearly half of PTB patients were clinically diagnosed by PTB only by manifestations, radiographic imaging and laboratory examination without an aetiological evidence, especially in low- and middle-income countries with constrained resources and a high PTB prevalence (Alavi-Naini et al., 2012; Gao, 2018; Ahmad et al., 2019).

The long cultivation time, high equipment requirements and unqualified sputum sample quality constitute important limitations of the diagnostic ability and use of pathogen−based detection, causing insufficient understanding and misdiagnosis of clinically diagnosed PTB (Yu et al., 2019). The resulting treatment delay and undertreatment represent important risk factors in the disease transmission and negatively affect the management of clinically diagnosed PTB (Hernandez-Garduno et al., 2004; Zhang et al., 2017). The recent recommendations of the WHO include non−pathogen−based detection to improve the identification of clinically diagnosed PTB using rapid and universal methods (Martinez and Andrews, 2019). Therefore, biomarkers of the host immune responses might provide key insights to solve this problem. Long non-coding RNA (lncRNA) is non-coding RNA with a length greater than 200 nucleotides. The change in the lncRNA is the earliest spatiotemporal event after the virus invasion to initiate the host response, which is the upstream event of proteomics and metabolomics and happens earlier than the generation of antibodies (Rossetti et al., 2013; Zhao et al., 2016; Tan et al., 2018). Accumulating evidence has indicated that lncRNA is closely associated with the occurrence or development of PTB and has the potential to be an early and noninvasive biomarker for clinically diagnosed PTB (Wang et al., 2015, 2019; Chen et al., 2017; Li et al., 2020). In a previous microarray analysis study of our research group, we showed that the expression of lncRNA n344917 (n344917 for short; located on chromosome 6: 85677082–85678394; lncRNA microarray data have been deposited in the Gene Expression Omnibus under accession no. GSE119143) was significantly down-regulated in the peripheral blood mononuclear cells (PBMC) of patients with clinically diagnosed PTB (FC = 2.5, *P* < 0.05). According to the NONCODE database, n344917 was highly expressed in both lymphocytes and leukocytes, which suggests that it may be associated with the immune response to *M. tuberculosis*. Specifically, it could be valuable to further investigate the application of n344917 in the identification of clinically diagnosed PTB.

Together with novel molecular biomarkers, many studies have focused on integrating laboratory and electronic medical records (EHR) variables in a combined prediction model. El-Solh et al. (1999) developed an artificial neural network model based on demographic variables, constitutional symptoms and radiographic findings, which can predict active PTB with a high accuracy (*c*-indices ± SEM = 0.947 ± 0.028). However, the value of this model in the clinically diagnosed PTB was not investigated. In addition, due to the limitations of the sample size, different populations and lack of external validation, it is difficult for many prediction models to precisely identify TB (Van Wyk et al., 2017). Many studies tried to find a possible solution to these problems. Cross et al. (2013) demonstrated that adding appropriate biomarker information could improve the accuracy of case reclassification by at least 10%. Therefore, we hypothesized that a prediction model based on effective lncRNA, laboratory and EHR variables can represent a promising rapid and universal method to enhance the identification of clinically diagnosed PTB.

In this study, we aimed to address the following research questions: (1) verifying the diagnostic value of the novel molecular biomarker of n344917; (2) developing and validating a rapid and universal prediction model in laboratory combining n344917, laboratory and EHR variables to identify clinically diagnosed PTB.

## Materials and Methods

### Study Protocol

**FIGURE 1:**
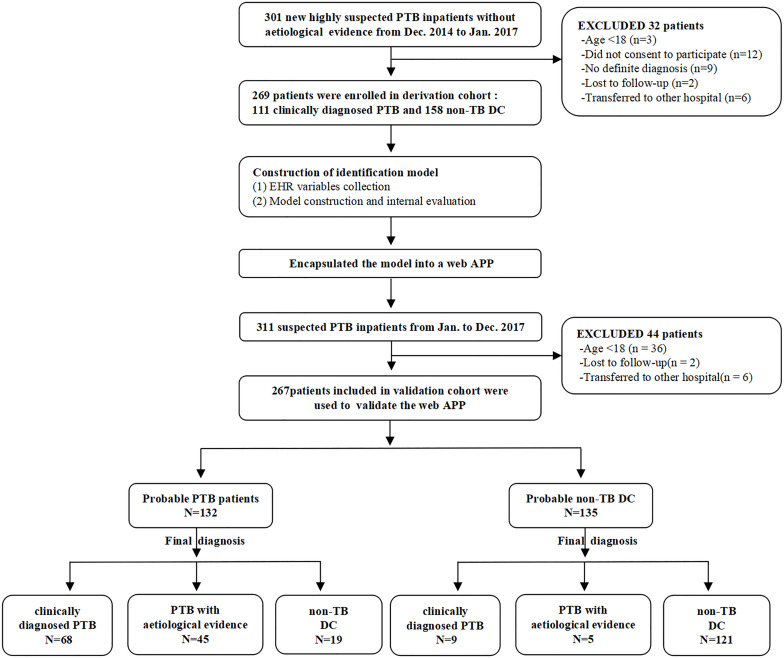
Study flow chart.

This research was designed to be performed in three steps. First, the expression level of n344917 selected by microarray was detected using reverse transcriptase quantitative real-time PCR in all participants for biomarker verification step. Next, in the modeling step, we used n344917, laboratory and EHR variables to extract the features and construct a prediction model in the derivation cohort. The internal 10-fold cross validation were used to test the model. Finally, during the external validation step, the optimal model was encapsulated as an open-source and free predictive web-based application. We evaluated it to further confirm that can produce good performance in an independent validation cohort. Figure 1 shows the flowchart of the study.

### Participants Recruitment

For the derivation cohort, we prospectively and consecutively recruited patients with a clinical suspicion of PTB without an aetiological evidence (smear microscopy, culture, or nucleic acid amplification test) who were admitted from Dec 2014 to Jan 2017 to the Respiratory and Infection Department of the West China Hospital, Sichuan University. The inclusion criteria included the following: (a) age ≥ 18 years; (b) clinical manifestations > 2 weeks and a disease history with high suspect of PTB. (c) at least two successive AFB sputum smears, one MTB-DNA PCR and one mycobacterium culture were all negative. (d) anti-tuberculosis therapy < 7 days on admission. Clinically diagnosed PTB and non-TB control patients were diagnosed following the Chinese diagnostic criteria for PTB by two experienced pulmonologists separately (The National Health and Family Planning Commission, WS 288–2017).

The independent validation cohort consisted of patients collected prospectively from Jan 2017 to Dec 2017 using a similar collection strategy. The difference was that clinically suspected PTB patients who eventually got an aetiological evidence were also enrolled to validate the generalization ability of the model.

### Ethics Statement

Informed consents were obtained from all the participants. This study was approved by the Clinical Trials and Biomedical Ethics Committee of West China [no. 2014 (198)] and was performed in accordance with the ethical standards as laid down in the 1964 Declaration of Helsinki and its later amendments or comparable ethical standards.

### LncRNA n344917 Expression Verification

The experimental scheme referred to the preceding exploration of our research group (Bai et al., 2019). Peripheral blood samples were collected from the participants and isolated to obtain PBMC. Total RNA was extracted according to the Trizol reagent specifications, and its concentration and quality were measured using a spectrophotometer. Then, the RNA was reverse transcribed into complementary DNA using the Takara Prime Script^TM^RT reagent Kit with gDNA Eraser (Takara, Japan). The expression level of n344917 was detected using qRT-PCR according to the SYBR method. The reaction system was as follows: 5 μL of Mix (2 × KAPA SYBR FAST qPCR Master Mix2 Universal), 0.2 μL of specific forward primers, 0.2 μL of specific reverse primers, and 1 μL of reverse transcripts.

### Laboratory and EHR Information Collection

Corresponding EHR (demographic, clinical manifestation, and radiological variables) and laboratory variables were collected on admission. The specific clinical manifestation included cough, expectoration, chest discomfort, hemoptysis, low-grade fever (37.4–38°C), weight loss (defined as a 10% reduction of an ideal body weight within 6 months), night sweats, poor appetite and fatigue. Medical imaging examination was performed by radiologists and the imaging characteristics associated with PTB (including polymorphic abnormality, calcification, cavities, bronchus sign, or hydrops on CT) were specifically evaluated. Laboratory variables, including complete blood count, coagulation function and biochemical examination, were collected through the laboratory management system of West China Hospital of Sichuan University and all the laboratory inspections were performed by qualified laboratory personnel in accordance with the standard operating procedure.

### Modeling

In the derivation cohort, the Least Absolute Shrinkage and Selection Operator (LASSO) regression analysis was used for variable selection, which is an important method in data fitting (Tibshirani, 1997). LASSO regression constructs a penalty function [penalty term: Sum (abs(b)) < = *t*] to compress the coefficients of the variables. The variables with a coefficient of 0 are eliminated, and a panel of optimal and representative variables are finally obtained. This can effectively avoid the influence of factors like the number of variables, different orders of magnitude, various units and possible co-linearity between the indicators on the classical analysis methods (Vreeman et al., 2015; Privé et al., 2019). In this regard, LASSO can enhance the generalization ability of the refined model. Then, the predictive model was constructed by incorporating the representative variables selected by LASSO into logistic regression. The model was then further optimized and internally validated through 10-fold cross-validation to select the final model.

### Web Application Construction and Independent Validation

The optimal model was encapsulated as an open-source and free predictive web-based application through the “shiny R” program package (Jimmy et al., 2016). Both clinicians and patients can enter the necessary information in the graphical user interface (GUI) and directly obtain the probability of PTB. The performance of the web application was assessed from several aspects in the validation cohort: (1) The accuracy was assessed according to the area under the curve (AUC). (2) The consistency was evaluated by calibration curves. (3) The Decision Curve Analysis (DCA) was used to estimate the net clinical benefits. (4) The Net Reclassification Improvement (NRI) and Integrated Discrimination Improvement (IDI) indexes were adopted to evaluate the improvement of the model diagnostic ability after the addition of n344917.

### Statistical Analysis

The continuous variables were represented by the median (upper and lower quartiles), and the categorical variables were represented by the frequency (percentage). The Mann–Whitney *U* test and chi-square test were used to analyze the continuous variables and categorical variables, respectively. LASSO was used for feature selection. Multivariate logistic regression was used to construct the identification model. The LASSO algorithm was implemented using the “glmmet” package, and the logistic regression model was established using the “glm” package. The web-based application was built using the “shiny” package of R. All statistical analyses were done using R version 3.5.0.

## Results

### Participants

A total of 536 PTB patients were finally included in this research. The mean of age groups in PTB and non-PTB groups was 38.87 (age range 18–81 years) and 57.29 (age range 18–89 years), respectively. The derivation cohort consisted of 269 cases (111 clinically diagnosed PTB and 158 non-TB DC), while the validation cohort consisted of 267 cases (77 clinically diagnosed PTB and 140 non-TB DC, 50 PTB with an aetiological evidence). The laboratory findings and EHR data of these patients are shown in Supplementary Table 1.

### Expression Levels of n344917

**FIGURE 2:**
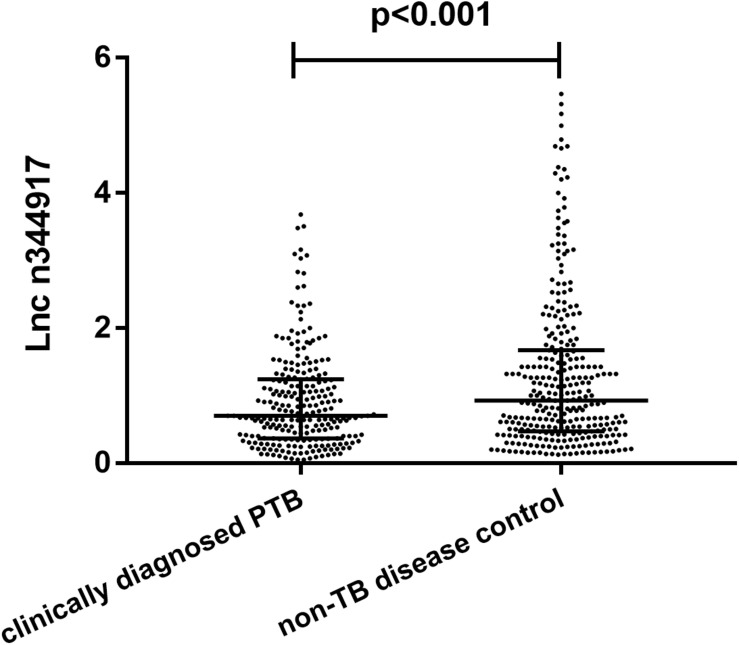
Relative expression levels of lncRNA n344917 in clinically diagnosed PTB and non-TB disease control.

Compared with the non-TB control subjects, n344917 was significantly down-regulated in the clinically diagnosed PTB patients (0.69 vs. 0.95, *p* < 0.001; Figure 2).

### Model Development

**FIGURE 3:**
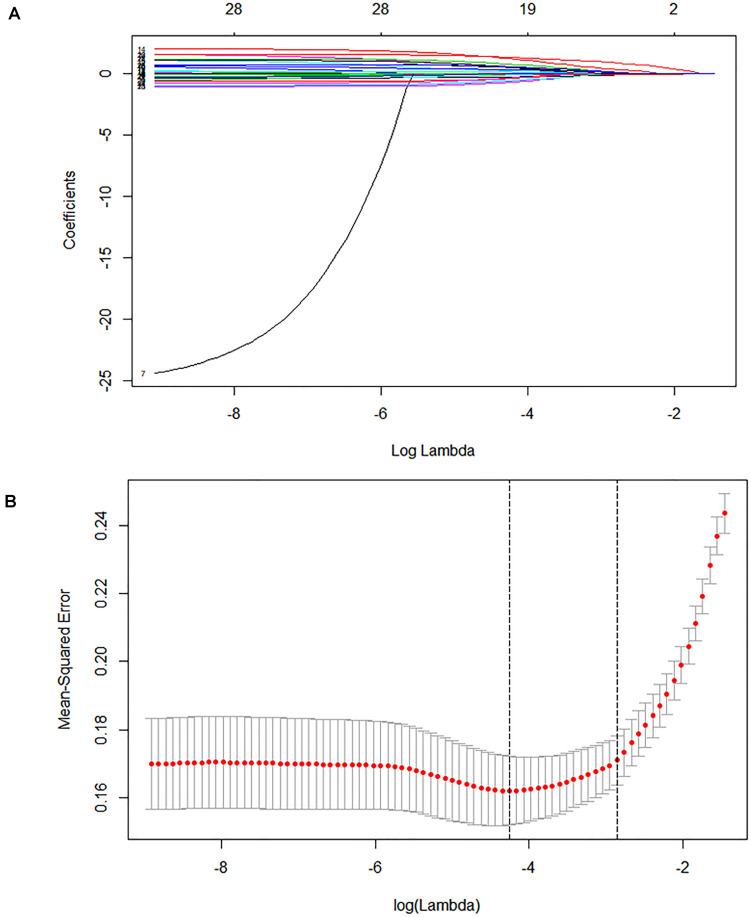
Lasso feature selection diagram. The *x*-coordinate is the logarithm function of the penalty coefficient λ, and the *y*-coordinate is the mean square error. **(A)** As lambda changes, the coefficient of the variable is compressed to zero. **(B)** The dotted line on the left represents the value of the λ log function with the minimum mean squared error, and the right represents the best lambda log function. The value at the top of the image is the number of features.

In the derivation cohort, seven variables [n34491, age, CT calcification, cough, TB-interferon gamma release assay (TB-IGRA), and low-grade fever and weight loss, as listed in Table 1] were selected by the LASSO algorithm as the predictors of clinically diagnosed PTB and included in the multivariate logistic regression model (Figure 3).

**TABLE 1:** Indicators in the web application.

Derivation cohort (269)				Validation cohort (217)		
	
	Non-TB DC (158)	Clinically diagnosed PTB (111)	*P*-value	Non-TB DC (140)	Clinically diagnosed PTB (77)	*P*-value
COUGH			0.023*			0.949
Negative	82 (51.90%)	42 (37.84%)		63 (45.00%)	35 (45.45%)	
Positive	76 (48.10%)	69 (62.16%)		77 (55.00%)	42 (54.55%)	
LOW-GRADE FEVER			0.001*			0.168
Negative	102 (64.56%)	50 (45.05%)		88 (62.86%)	41 (53.25%)	
Positive	56 (35.44%)	61 (54.95%)		52 (37.14%)	36 (46.75%)	
WEIGHT LOSS			<0.001*			<0.001*
Negative	144 (91.14%)	82 (73.87%)		134 (95.71%)	59 (76.62%)	
Positive	14 (8.86%)	29 (26.13%)		6 (4.29%)	18 (23.38%)	
CT CALCIFICATION			<0.001*			0.001*
Negative	109 (68.99%)	44 (39.64%)		86 (61.43%)	30 (38.96%)	
Positive	49 (31.01%)	67 (60.36%)		54 (38.57%)	47 (61.04%)	
TB-IGRA			<0.001*			<0.001*
Negative	103 (65.19%)	25 (22.52%)		97 (69.29%)	19 (24.68%)	
Positive	55 (34.81%)	86 (77.48%)		43 (30.71%)	58 (75.32%)	
AGE	58 (47,66)	36 (24.0,50.5)	<0.0001*	60 (46,70)	37 (22,52)	<0.0001*
N344917	0.91 (0.50,1.74)	0.66 (0.36,1.22)	<0.0001*	1.00 (0.43,1.67)	0.74 (0.35,1.24)	0.0005*

Data are presented as *n* (%) for categorical variables and as median (upper and lower quartiles) for continuous variables. *P value < 0.05.

The risk of clinically diagnosed PTB was calculated as follows:

Risk=1/(1+exp(-(0.4674133-0.4849920*n344917

-0.0543645*a⁢g⁢e+1.0900268*C⁢T⁢c⁢a⁢l⁢c⁢i⁢f⁢i⁢c⁢a⁢t⁢i⁢o⁢n

+0.8190174*cough+1.6366705*TBIGRA+0.7601317*

(1) lowgradefever+0.6216830*loss))),(cutoff=0.657).

### Web Application and Independent Validation

**FIGURE 4:**
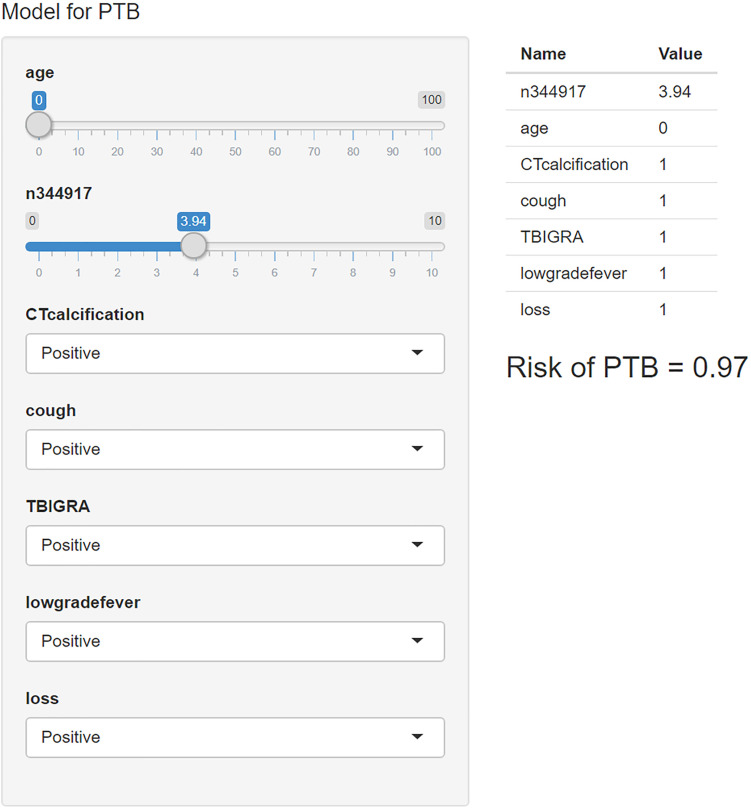
Schematic illustration of the web application. We entered the corresponding parameters into the web application according to the laboratory information and clinical symptoms. Then, the model showed the probability of PTB.

We used the “R shiny” package to provide a visual and operational GUI for this model, where the user can directly obtain the prediction probability by entering or selecting a variable in the web-based application (https://ziruinptb.shinyapps.io/shiny/; Figure 4).

**FIGURE 5:**
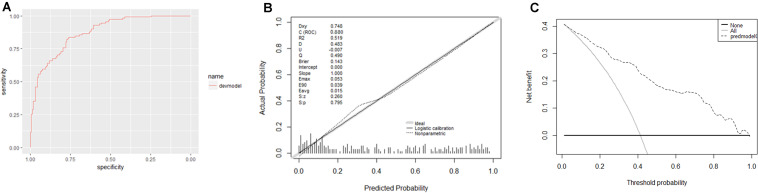
**(A)** Receiver operator characteristic curve. The AUC was 0.88 in the derivation cohort. **(B)** Calibration curves. The 45° shaded line represents the ideal prediction, and the prediction probability is consistent with the actual observation probability. The blue line represents the actual prediction of the model. The stapled histogram on the bottom line represents the distribution of the patients’ predicted probability. Abbreviations: Dxy = Somer’s D rank correlation, R2 = Nagelkerke-Cox-Snell-Maddala-Magee R-squared index, D = Discrimination index, U = Unreliability index, Q = Quality index, Emax = maximum absolute difference in the predicted and calibrated propabilities, S:z = Spiegelhalter *Z*-test, and S:p = two-tailed *p*-value of the Spiegelhalter *Z*-test. **(C)** Decision analysis curve. The horizontal axis is the threshold probability of the PTB occurrence. The vertical axis shows the clinical benefits that the patients may gain or lose using the web application. Dotted line: prediction model. Solid line: all patients were PTB. Horizontal line: all patients were not PTB.

Furthermore, this study included 267 patients (77 clinically diagnosed PTB and 140 non-TB DC, 50 PTB with an aetiological evidence) in an independent validation cohort for evaluating the performance of the web-based application (Figure 1). The NRI and IDI indexes were 0.121 and 0.1103, respectively, (*P* < 0.05), indicating that the predictive performance was significantly improved after adding n344917. The comparison between the prediction results and the follow-up results showed that the model has a sensitivity of 88.98%, a specificity of 86.43%, a positive predictive value of 85.61%, and a negative predictive value of 89.63%; plus, the AUC value was 0.88 (Figure 5A). The calibration curve showed the deviation between the fitting curve and the actual curve to be insignificant (Figure 5B). In the DCA (Figure 5C), the vertical axis is the net benefit, while the horizontal axis is the probability threshold. The curve with the highest net benefit may offer the optimal treatment choice and can assist clinicians in making appropriate therapeutic decisions. These findings indicate that the web application exhibits high net benefits when the threshold probability falls between 20 and 80%.

## Discussion

Etiological detection is the most important basis for the PTB diagnosis (Ahmad et al., 2019). Nevertheless, due to the insufficient sensitivity and timeliness of sputum smear microscopy and culture methods, the true positive rate is only about 30–50% (Alavi-Naini et al., 2012; Liu et al., 2018). Symptoms are often the most important basis for clinically diagnosed PTB, but this results in low sensitivity and specificity and can lead to misdiagnosis (Walzl et al., 2018; Wu et al., 2018). So far, there has been little discussion to improve the diagnosis of clinically diagnosed PTB and less attempted to incorporate new molecular predictors, which always have an insufficient sample size (TB sample size range: 22–114) and without an independent validation cohort (Chen et al., 2017; He et al., 2017; Shih et al., 2019; Wang et al., 2019; Li et al., 2020).

To some extent, our prediction model avoids these deficiencies and has the advantages of prospective, relatively sufficient sample size, satisfactory performance and independent validation cohort validation. In this study, we establish a new approach to assist the identification of PTB patients via three main steps. First, the candidate biomarker, lncRNA n344917, is confirmed to be down-regulated in clinically diagnosed PTB. Second, a novel rapid universal and comprehensive prediction model combining the molecular biomarker, EHR and laboratory variables, is constructed. This will make up for the deficiency of the detection method and enhance the identification of clinically diagnosed PTB from more aspects. Third, the prediction model is encapsulated as a user-friendly web-based application and externally validated.

The results confirm the significant differential expression of n344917 in the clinically diagnosed PTB tuberculosis (*P* < 0.001). Next, we use the LASSO algorithm to comprehensively analyze 35 variables including the biomarker, EHR and laboratory variables. In the past, many researchers believed that clinical indicators with significant statistical differences may be closely related to the disease and can be used to construct diagnostic models. However, studies have increasingly found that it is not objective and may result in wasting data resources and even misleading decisions (Amrhein et al., 2019; Griffiths and Needleman, 2019). Independent of the variable selection based on statistical significance, LASSO shrinks the coefficients of the variables by regularization to select and obtain an effective and concise set of variables with coefficients greater than 0 (Vreeman et al., 2015). This method can effectively avoid over-fitting based on the significance difference and prevent the influence of factors like the number of variables, different orders of magnitude, various units and possible co-linearity between the indicators in the classical analysis methods, which ensures the stability of the model when new samples are used for verification (Privé et al., 2019). Finally, a panel of optimal and representative variables are obtained (n344917 expression, age, CT calcification, cough, TB-IGRA, and low-grade fever and weight loss). Among these predictors, n344917 still plays an important role in the model [NRI and IDI indexes are 0.121 and 0.1103, respectively, (*P* < 0.05)].

Verification results show that the performance of our model still has a compelling predictive effect in the independent validation cohort, including PTB patients with an aetiological evidence, clinically diagnosed PTB and non-TB DC, which indicates the reliability, robustness and broad universality of our model. Relatively high AUC and calibration curve with a good fitting effect indicate that the model has good discrimination and consistency. The DCA curve demonstrates that the prediction of our model has relatively high net benefits when the threshold probability falls between 20 and 80%. DCA was first invented by Vickers and co-workers in 2006 and has been widely used to assess the net benefits of diagnostic testing, as well as the impact of under- and over-treatment (Vickers and Elkin, 2006; Van Calster et al., 2018). Given that DCA is more concerned with the consequences of predictive information and may complement the deficiency of previous evaluation that only focuses on accuracy, it has been recommended by top clinical journals and used in various clinical fields (Vickers et al., 2008; Fitzgerald et al., 2015). In addition, the sensitivity and specificity of our prediction model are 88.89 and 86.43%, while the positive predictive value is 85.61% and negative predictive value is 89.63%. Hence, through the earlier anti-tuberculosis treatment, given according to the diagnostic results of our proposed model, it is hoped that the disease damage and infection rate can be effectively reduced, thereby ultimately ending PTB transmission globally.

Several studies have documented that diagnostic applications have great potential in providing diagnostic definitions. Therefore, the model is encapsulated as an open-source and free predictive web-based application for the actual clinical application. It is free, user-friendly and relatively simple, such that it does not need any programming foundation to be operated. Both clinicians and patients can directly obtain the probability of PTB by entering the necessary information as input, providing effective support for automated medical diagnosis, especially in low- and middle-income countries with constrained resources and high prevalence of PTB. It can also help doctors to compare the results with other information in order to reach a comprehensive conclusion, which represents an enhancement for assessing difficult-to-diagnose PTB to medical diagnosis. With the popularization of digital medical and mobile terminals, we believe that our research can provide better diagnostic services now and in the future.

## Conclusion

In conclusion, this study reveals lncRNA n344917 as a potential molecular biomarker for the clinically diagnosed PTB. We constructed and validated a prediction model combining n344917, laboratory and EHR variables to enhance the identification of PTB and encapsulated it as an open-source and free predictive web-based application for the actual clinical application. In the future, we plan to optimize and validate our model among different ethnic groups. Achieving better understanding of the biological mechanisms underlying the association between n344917 and disease development and progression will be needed to improve the early diagnosis of PTB.

## Data Availability Statement

The datasets presented in this study can be found in online repositories. The names of the repository/repositories and accession number(s) can be found in the article/Supplementary Material.

## Ethics Statement

The studies involving human participants were reviewed and approved by Clinical Trials and Biomedical Ethics Committee of West China. The patients/participants provided their written informed consent to participate in this study.

## Author Contributions

BY and CT had full access to all the data in the study, were responsible for the integrity of the data, and supervised the work. ZM, MW, SG, and YZ conceptualized and designed the experiment, and analyzed or interpreted the data. ZM and MW drafted the manuscript. BY and XH critically revised the manuscript for important intellectual content. ML, XH, HB, and QW collected the data. BY obtained funding. All authors contributed to the article and approved the submitted version.

## Disclaimer

The authors would like to express their gratitude to EditSprings (https://www.editsprings.com/) for the expert linguistic services provided and also to all individuals who participated in or helped with this research project.

## Conflict of Interest

The authors declare that the research was conducted in the absence of any commercial or financial relationships that could be construed as a potential conflict of interest.
