# Supplementary material for: Novel Long Non-coding RNA and LASSO Prediction Model to Better Identify Pulmonary Tuberculosis: A Case-Control Study in China
Source: Front Mol Biosci. 2021 May 25;8:632185. doi: 10.3389/fmolb.2021.632185 (PMC8185277; doi:10.3389/fmolb.2021.632185)
Supplement: Supplementary file 1 [file Table_1.DOCX]

**Supplementary 1. Comparison of n344917 and EHR indicators**

|  | Derivation cohort(269) | | | Validation cohort(217) | | |
| --- | --- | --- | --- | --- | --- | --- |
|  | Non-TB DC (158) | Clinically diagnosed PTB(111) | P-value | Non-TB DC (140) | Clinically diagnosed PTB(77) | P-value |
| Sex | |  | 0.969 |  |  | 0.683 |
| male | 95 (60.13%) | 67 (60.36%) |  | 87 (62.14%) | 50 (64.94%) |  |
| female | 63 (39.87%) | 44 (39.64%) |  | 53 (37.86%) | 27 (35.06%) |  |
| Cough | |  | 0.023 |  |  | 0.949 |
| negative | 82 (51.90%) | 42 (37.84%) |  | 63 (45.00%) | 35 (45.45%) |  |
| positive | 76 (48.10%) | 69 (62.16%) |  | 77 (55.00%) | 42 (54.55%) |  |
| Expectoration | |  | 0.073 |  |  | 0.945 |
| negative | 121 (76.58%) | 74 (66.67%) |  | 97 (69.29%) | 53 (68.83%) |  |
| positive | 37 (23.42%) | 37 (33.33%) |  | 43 (30.71%) | 24 (31.17%) |  |
| Cheat discomfort | |  | 0.152 |  |  | 0.224 |
| negative | 126 (79.75%) | 96 (86.49%) |  | 123 (87.86%) | 63 (81.82%) |  |
| positive | 32 (20.25%) | 15 (13.51%) |  | 17 (12.14%) | 14 (18.18%) |  |
| Hemoptysis | |  | 0.179 |  |  | 0.029 |
| negative | 147 (93.04%) | 98 (88.29%) |  | 131 (93.57%) | 65 (84.42%) |  |
| positive | 11 (6.96%) | 13 (11.71%) |  | 9 (6.43%) | 12 (15.58%) |  |
| Low-grade fever | |  | 0.001 |  |  | 0.168 |
| negative | 102 (64.56%) | 50 (45.05%) |  | 88 (62.86%) | 41 (53.25%) |  |
| positive | 56 (35.44%) | 61 (54.95%) |  | 52 (37.14%) | 36 (46.75%) |  |
| Weight loss | |  | <0.001 |  |  | <0.001 |
| negative | 144 (91.14%) | 82 (73.87%) |  | 134 (95.71%) | 59 (76.62%) |  |
| positive | 14 (8.86%) | 29 (26.13%) |  | 6 (4.29%) | 18 (23.38%) |  |
| Night sweat | |  | 0.275 |  |  | 0.047 |
| negative | 95 (60.13%) | 74 (66.67%) |  | 77 (55.00%) | 53 (68.83%) |  |
| positive | 63 (39.87%) | 37 (33.33%) |  | 63 (45.00%) | 24 (31.17%) |  |
| Poor appetite | |  | 0.97 |  |  | 0.312 |
| negative | 100 (63.29%) | 70 (63.06%) |  | 95 (67.86%) | 47 (61.04%) |  |
| positive | 58 (36.71%) | 41 (36.94%) |  | 45 (32.14%) | 30 (38.96%) |  |
| Fatigue | |  | 0.816 |  |  | 0.868 |
| negative | 96 (60.76%) | 69 (62.16%) |  | 78 (55.71%) | 42 (54.55%) |  |
| positive | 62 (39.24%) | 42 (37.84%) |  | 62 (44.29%) | 35 (45.45%) |  |
| CT calcification | |  | <0.001 |  |  | 0.001 |
| negative | 109 (68.99%) | 44 (39.64%) |  | 86 (61.43%) | 30 (38.96%) |  |
| positive | 49 (31.01%) | 67 (60.36%) |  | 54 (38.57%) | 47 (61.04%) |  |
| CT cavity | |  | 0.024 |  |  | 0.114 |
| negative | 119 (75.32%) | 96 (86.49%) |  | 99 (70.71%) | 62 (80.52%) |  |
| positive | 39 (24.68%) | 15 (13.51%) |  | 41 (29.29%) | 15 (19.48%) |  |
| CT hydrops | |  | 0.078 |  |  | 0.222 |
| negative | 49 (31.01%) | 46 (41.44%) |  | 50 (35.71%) | 34 (44.16%) |  |
| positive | 109 (68.99%) | 65 (58.56%) |  | 90 (64.29%) | 43 (55.84%) |  |
| CT bronchus sign | |  | 0.002 |  |  | 0.002 |
| negative | 152 (96.20%) | 95 (85.59%) |  | 134 (95.71%) | 64 (83.12%) |  |
| positive | 6 (3.80%) | 16 (14.41%) |  | 6 (4.29%) | 13 (16.88%) |  |
| CT polymorphic abnormality | |  | 0.439 |  |  | 0.685 |
| negative | 73 (46.20%) | 46 (41.44%) |  | 64 (45.71%) | 33 (42.86%) |  |
| positive | 85 (53.80%) | 65 (58.56%) |  | 76 (54.29%) | 44 (57.14%) |  |
| TB-IGRA | |  | <0.001 |  |  | <0.001 |
| negative | 103 (65.19%) | 25 (22.52%) |  | 97 (69.29%) | 19 (24.68%) |  |
| positive | 55 (34.81%) | 86 (77.48%) |  | 43 (30.71%) | 58 (75.32%) |  |
| N344917 | 0.91(0.50,1.74) | 0.66(0.36,1.22) | <0.0001 | 1.00(0.43,1.67) | 0.74(0.35,1.24) | 0.0005 |
| AGE | 58(47,66) | 36(24.0,50.5) | <0.0001 | 60(46,70) | 37(22,52) | <0.0001 |
| ALB | 37.45(32.05,41.73) | 37.40(32.75,41.30) | 0.9618 | 37.45(31.70,41.32) | 37.7(33.2,41.6) | 0.6137 |
| GLOBIN | 29.40(25.75,33.15) | 30.00(26.35,35.70) | 0.0692 | 28.95(24.88,33.02) | 29.30(25.50,33.60) | 0.7016 |
| RBC | 4.02(3.48,4.51) | 4.26(3.92,4.83) | 0.0002 | 3.86(3.37,4.54) | 4.44(3.98,4.97) | <0.0001 |
| HB | 118(100,132.5) | 125(108.5,140.5) | 0.003 | 113(96,129.25) | 127(116,137) | 0.0003 |
| HCT | 0.37(0.31,0.41) | 0.38(0.33,0.42) | 0.0401 | 0.36(0.31,0.40) | 0.39(0.34,0.42) | 0.0022 |
| PLT | 231.0(146.5,299.0) | 237(177,305) | 0.2068 | 199.5(141,290.25) | 233(167,318) | 0.0568 |
| WBC | 6.30(4.54,8.55) | 6.05(4.89,8.25) | 0.8204 | 7.31(5.45,9.70) | 5.98(4.44,8.47) | 0.0161 |
| N | 4.05(2.71,6.24) | 4.04(3.23,5.80) | 0.6757 | 4.97(3.25,6.83) | 4.07(2.61,5.70) | 0.0584 |
| L | 1.27(0.86,1.74) | 1.15(0.82,1.51) | 0.3354 | 1.34(0.96,1.82) | 1.22(0.88,1.61) | 0.1001 |
| M | 0.40(0.25,0.570) | 0.48(0.35,0.67） | 0.032 | 0.48(0.34,0.72) | 0.43(0.31,0.64) | 0.0308 |
| CRP | 18.00(7.45,58.10) | 18.00(5.72,62.35) | 0.2445 | 18.60(6.49,70.90) | 11.80(3.26,40.70) | 0.0642 |

TB-IGRA, TB-interferon gamma release assay; RBC, red blood cell; Hb, Hemoglobin; Hct, hematocrit;PLT, Platelets; WBC, white blood cell;ALB, albumin; N, neutrophils; L, lymphocytes; M, monocytes; CRP, C-reactive protein
